# Identification of mutations in *DYNC2LI1*, a member of the mammalian cytoplasmic dynein 2 complex, expands the clinical spectrum of Jeune/ATD ciliopathies

**DOI:** 10.1186/2046-2530-4-S1-P59

**Published:** 2015-07-13

**Authors:** K Kessler, N Falk, I Wunderlich, M Fröhlich, N Hauer, A Gießl, JH Brandstätter, H Sticht, AB Ekici, S Uebe, E Seemanová, A Reis, R Roepman, C Thiel

**Affiliations:** 1 Institute of Human Genetics, Friedrich-Alexander-Universität Erlangen-Nürnberg, Erlangen, Germany; 2 Department of Biology, Animal Physiology, Friedrich-Alexander-Universität Erlangen-Nürnberg, Erlangen, Germany; 3 Institute of Biochemistry, Friedrich-Alexander-Universität Erlangen-Nürnberg, Erlangen, Germany; 4 Department of Clinical Genetics, Institute of Biology and Medical Genetics, 2nd Medical School, Charles University, Prague, Czech Republic; 5 Department of Human Genetics, Radboud University Medical Center, Nijmegen, The Netherlands

## 

Ciliopathies are caused by defects in formation, maintenance and function of the primary cilium and underlying genes affect the dynein motor, intraflagellar transport complexes, or the basal body. In a patient of non-consanguineous parents presenting an intermediate phenotype between asphyxiating thoracic dystrophy and Ellis-van Crefeld syndrome we performed exome sequencing. Variants were selected based on potential ciliary function as identified in a yeast two-hybrid screen with NEK1, a basal body protein involved in short rib-polydactyly type Majeweski (SRPSII). We identified compound heterozygous nonsense (p.R208X) and missense (p.T221I) mutations in *DYNC2LI1 *segregating in the family. DYNC2LI1 is ubiquitously expressed and interacts with DYNC2H1 to form the dynein 2 complex important for retrograde intraflagellar transport. The hypothetical protein caused by the nonsense mutation lacks the coiled-coil domain involved in protein interaction and dimerization. The mutation p.T221I affects a highly conserved nucleoside triphosphate hydrolase domain responsible for GTPase driven dynein protein localization. Mutations in both *DYNC2LI1 *interacting partners *DYNC2H1 *and *NEK1 *are associated with ATD and SRPSs. We screened further patients of our short stature cohort and identified in two siblings heterozygous mutations in *DYNC2LI1 *(p.M1T) and its interaction partner *DYNC2H1 *(p.K495T). The DYNC2H1 mutation was previously reported by El Hokayem et al. compound heterozygous with a splice site mutation in a patient with SRPSII. Our results might indicate a possible digenic diallelic inheritance in our patients. This is the first report of mutations in *DYNC2LI1 *as part of the dynein 2 complex further expanding the clinical spectrum of ciliopathies.

